# AURKB activates EMT through PI3K/AKT signaling axis to promote ICC progression

**DOI:** 10.1007/s12672-023-00707-1

**Published:** 2023-06-15

**Authors:** Peng Ma, Ying Hao, Wei Wang, Yue-Feng Zhang, Kai-Huan Yu, Wei-Xing Wang

**Affiliations:** Deportment of Hepatobiliary Surgery, Renmin Hospital, Wuhan University, Wuhan, 430060 Hubei Province People’s Republic of China

## Abstract

Intrahepatic cholangiocarcinoma (ICC) is a fatal disease and the molecular mechanism of its progression remains unknown. Aurora Kinase B (AURKB) is a central regulator of chromosome separation and cytokinesis and is abnormally expressed in a variety of cancer cells. This research aimed to explore the effect of AURKB in occurrence and metastasis of ICC. We found that AURKB showed a progressive up-regulation pattern from normal bile duct tissue to ICC with high invasion. Our data showed that AURKB significantly promoted ICC cell proliferation, induced epithelial-mesenchymal transition (EMT), migration and invasion through gain- and loss- of function experiments. In *vivo* results consistently showed that AURKB up-regulation not only promoted tumor growth, but also promoted tumor metastasis. Importantly, we discovered that AURKB regulates the expressions of EMT-related genes via PI3K/AKT signaling axis. Herein, our results suggest that AURKB induced EMT through the activation of PI3K/AKT signaling pathway is critical to the progression of ICC, which may be a prospective therapeutic treatment for overcoming ICC metastasis and progression.

**Supplementary Information:**

The online version contains supplementary material available at 10.1007/s12672-023-00707-1.

## Introduction

Intrahepatic cholangiocarcinoma (ICC) is the second most common primary liver malignancy, accounting for about 10–15% of all primary liver cancers, and its incidence is on the rise [1, 2]. Surgical excision is currently the only viable treatment option for ICC. While ICC is generally asymptomatic in the early stages, most patients with ICC are diagnosed with advanced stages, and resectable and curable remains low. The high aggressiveness of ICC makes the tumor prone to multifocal, lymph node metastasis, and vascular invasion, with 60% of patients undergoing surgery developing recurrent or metastatic disease [3, 4], resulting in poor long-term survival after resection [5]. Therefore, it is necessary to better investigate the potential molecular mechanism of the invasive and metastatic properties of ICC and search for new therapeutic targets to inhibit metastasis in order to develop better treatment strategies for this deadly cancer.

Aurora Kinase B (AURKB) is a serine/threonine kinase,which is known for its effect as a central regulator of chromosome separation and cytokinesis[6, 7], and is overexpressed in some tumors, such as lung, breast, pancreatic, ovarian, and prostate tumors [8–11]. Hence, AURKB may also contribute to tumor progression. For example, as a prognostic biomarker for gastric cancer, AURKB promotes the progression of gastric cancer through epigenetic activation of CCND1 expression [12]. Similar to the function of AURKB in mitosis, it is expected that the changes of AURKB amplification or overexpression could promote the proliferation of cancer cells. Compared with healthy controls, AURKB overexpression in prostate cancer tissues was increased, which was directly related to the malignancy of prostate cancer, and in *vitro* experiments confirmed that AURKB affected the proliferation level of prostate cancer cells [13]. Moreover, the expression of AURKB mRNA was also obviously elevated in hepatocellular carcinoma tissue compared with the healthy one, and it was found to be an independent marker of tumor aggressiveness and prognosis [14]. AURKB overexpression in tumors indicated that AURKB may be the potential target of tumors treatment. However, the function of AURKB in ICC is still unknown.

This study unveiled the role of AURKB in the progression of ICC and identified the underlying mechanism. There was a highly expression of AURKB in human ICC tissues and ICC cell lines. AURKB promotes tumor development and growth both in *vivo* and in *vitro*. Tumor metastasis is always the main cause of death in patients with malignant tumors. Epithelial-mesenchymal transition (EMT) is the key step that leads to the escape of metastatic tumor cells from the primary site. The main characteristics of EMT are to inhibit the expression of E-cadherin, promote the expression of N-cadherin and Vimentin, lose the polarized tissue of epithelial cells, and regulate the ability to migrate and invade [15]. We also found that AURKB modulate the related markers of EMT in ICC. In addition, other studies also showed that EMT play a crucial effect in the metastasis of ICC cells [16, 17]. Finally, our study demonstrated that AURKB promote ICC EMT. Mechanistically, AURKB induces EMT in ICC cells by activating PI3K/AKT pathway. The expression changes of E-cadherin, N-cadherin and Vimentin in HCCC9810 cells with low expression of AURKB and AURKB overexpressed RBE cells were reversed by AKT agonist SC-79 and AKT inhibitor MK2206, respectively. Taken together, these data identified that the AURKB—PI3K/AKT axis is critical for ICC EMT and development, suggesting that AURKB and AKT can be used as curative targets to surmount the poor prognosis in ICC patients with high AURKB expression.

## Materials and methods

### Clinical samples and cell cultures

From September 2017 to December 2018, 27 ICC specimens and paired adjacent normal bile duct specimens were obtained from Renmin Hospital of Wuhan University. After isolation of specimens, part of ICC tissues and matched adjacent normal liver tissue (tumor margin ≥ 1 cm) were immediately frozen in liquid nitrogen tank. The main exclusion criteria were other malignancies and neoadjuvant therapy. This research was approved by the Ethics Committee of Renmin Hospital of Wuhan University. The tissue samples used in this research were obtained with the patient's written informed consent. Tumors were classified according to the eighth edition of the Tumor Lymph Node Metastasis (TNM) classification system published by the International Union Against Cancer. Tumor differentiation followed the classification proposed by Edmondson and Steiner. Detailed clinicopathologic features of the 103 ICC specimens used in this study are listed in supplemental Table 1.

**Table 1:** Correlation between AURKB expression and clinicopathological parameters of 103 patients enrolled

Clinical characteristics		AURKB low (n = 53)	AURKB high (n = 50)	*P*
Age	≤ 55	24	22	0.896
	> 55	29	28	
Gender	Female	20	18	0.855
	Male	33	32	
HBV infection	Negative	15	17	0.532
	Positive	38	33	
HCV infection	Negative	46	45	0.612
	Positive	7	5	
Hepatolithiasis	Negative	38	32	0.403
	Positive	15	18	
Diabetes mellitus	Negative	41	33	0.200
	Positive	12	17	
Liver cirrhosis	No	8	10	0.512
	Yes	45	40	
ALT, U/L	≤ 40	43	33	0.081
	> 40	10	17	
CA19-9, ng/ml	≤ 37	29	14	**0.006**
	> 37	24	36	
Tumor number	Single	45	31	**0.008**
	Multiple	8	19	
Tumor size, cm	≤ 5	44	40	0.693
	> 5	9	10	
Tumor differentiation	I-II	26	31	0.187
	III-IV	27	19	
Micro vascular invasion	No	40	31	0.140
	Yes	13	19	
Lymph node metastasis	No	39	26	**0.023**
	Yes	14	24	
TNM stage	I	28	18	0.086
	II-III	25	32	

ALT, alanine aminotransferase; CA19-9, carbohydrate antigen 19–9

ICC cell lines, including RBE, HCCC9810, HuH28, HuCCT1, and CCLP1, were cultivated in RPMI 1640 medium with 10% fetal bovine serum (FBS) and 1% penicillin–streptomycin solution in a 5% CO_2_ incubator at 37 °C.

### Gene knockdown or overexpression of lentivirus transduction

The lentiviral construct for shRNA mediated AURKB silencing and AURKB overexpression was purchased from Shanghai GenePharma Co., Ltd., China. HCCC9810 cells were infected with pLKO.1 lentiviral vector to knockdown AURKB. The AURKB shRNA vector sequence was as follows: shAURBK1 (shRNA1): 5′CCGGCATCGTCAAGGTGGACCTAAACTCGAGTGTCACACTTAAGC ATCTCCTTTTTG-3′; shAURBK2 (shRNA2): 5′-CCGGGCAGAGAGATCGAAATCCAGGCTCGAG CCTGGATTTCGATCTCTCTGCTTTTTG-3′; shAURBK3 (shRNA3): 5′-CCGGGGAGTTGGCAGA TGCTCTAATCTCGAGATTAGAGCATCTGCCAACTCCTTTTTG-3′.

AURKB was overexpressed using pLVX lentiviral vector, which was cloned by the full-length CDS region of AURKB, and the pLVX lentiviral vector was used to infect RBE cells for AURKB overexpression. Stable transfected cells were selected in medium containing 5 mg/ml purinamycin for 7 days after 72 h transfection with lentivirus, and the infection efficiency was verified by qRT-PCR and western blotting.

### Immunohistochemical (IHC) analysis

IHC detected AURKB protein expression in ICC clinical specimens as described previously [18]. The tissues were fixed with 4% paraformaldehyde, embedded in paraffin, and sliced 3 μm. The anti-AURKB antibody (1:200: CST 28711S) was then used overnight at 4 ℃. The secondary antibody was cultured and stained with DAB reagent.

### Western blotting

RIPA buffer (Beyotime, China) was used to extract total proteins from cells. SDS-PAGE isolated the proteins and transferred to PVDF membrane. The membrane was blocked and then primary antibody was applied: AURKB (CST 28711S 1:1000); E-cadherin (CST 3915S 1:1000); N-cadherin (CST 13116S 1:1000); Vimentin (CST 5741S 1:1000); Snail (CST 3879S 1:1000); Snail (CST 3879 s 1:1000); Twist (CST 69366S 1:1000); GAPDH (CST 5174S 1:1000); p-AKT (CST 4060S 1:1000); t-AKT (CST 4691S 1:1000); p-ERK (CST 4370S 1:1000); t-ERK (CST 4695S 1:1000); p-P65 (CST 3033S 1:1000); t-p65 (CST 8242S 1:1000); p-JNK (CST 4668S 1:1000); t-JNK (CST 9252S 1:1000). The membranes were cleaned with TBST buffer solution and then incubated with secondary antibodies. The analysis was performed using the ECL kit (biosharp, China).

### Cell proliferation assay

Cell proliferation was detected using the CCK-8 cell viability assay kit (Beyotime, China). The cells were cultivated in 96-well plates at a concentration of 2 × 10^3^ cells/well for 24, 48, 72, 96 h. A total of 10 ul of CCK8 reagent was added to each well and incubated at 37 °C for 1 h. The 96-well plate was put into the enzyme label according to the program, and the wavelength of 450 nm was selected to measure the absorbance value.

### Colony formation experiment

The colony formation experiment was performed as described previously [19]. Briefly, cells (1000 per well) are planted into the 6-well plate. After 10 days, the colonies were fixed, stained and counted.

### Invasion assay

The invasion assay was performed as described previously [20]. Cells in 5 fields were randomly counted using an inverted light microscope.

### Wound healing experiment

The wound healing experiment was as described in previous study [21]. Taking images were obtained with an inverted optical microscope (Olympus) at 0 and 48 h time points.

### Cell cycle analysis

The cells were immobilized overnight in 70% ethanol. 24 h later, the cells were washed with 1 × PBS solution and centrifuged, then suspended in 1 × PBS solution and incubated with RNaseA at 37℃ for 30 min. Finally, the cells were stained with propyl iodide and analyzed by FACSCalibur system (BD Biosciences).

### Quantitative real-time polymerase chain reaction (qRT-PCR)

Total RNA was isolated from cells using Trizol (Thermo Fisher Scientific, USA). The qRT-PCR method is described in the previous study [22]. N-cadherin, Vimentin, E-cadherin, Snail, and Twist are normalized for GAPDH. 2^−ΔΔ^CT was used to analyze RNA expression levels. Primer sequences used in this study are shown as follows: E-cadherin: 5′-TACGCCTGGGACTCCACCTA-3′ (F), 5′-CCAGAAACGGAGGCCTGAT-3′ (R); N-cadherin: 5′-ATCCTACTGGACGGTTCG-3′ (F), 5′-TTGGCTAATGGCACTTGA-3′ (R); Vimentin: 5′-GAACGCCAGATGCGTGAAATG-3′ (F), 5′′-CCAGAGGGAGTGAATCCAGATTA-3′ (R); GAPDH: 5′-GTCATCCAACGGGAATGCA-3′ (F), 5′-TGATCGGTTACCGTGATCAAAA-3′ (R). Snail: 5′- TCGGAAGCCTAACTACAGCGA-3′ (F), 5′-AGATGAGCATTGGCAGCGAG-3′ (R). Twist: 5′- GTCCGCAGTCTTACGAGGAG-3′ (F), 5′-GCTTGAGGGTCTGAATCTTGCT-3′ (R). AKT: 5′-GAAGACGGGAGCAGGCG-3′ (F), 5′-ATCCTGGGACAGGGCACA-3′ (R); ERK (MAPK1): 5′-CGAGTGACGAGCCCATCG-3′ (F) GACCAGGGGTCAAGAACTGG -3′ (R); JNK (MAPK9): 5′-GGGAGAGCTGGTGAAAGGTT-3′ (F), 5′-CAGATCTCTGGCTTGACTTGTTTT-3′ (R); NF-kB (RELA): 5′-GCCGGGATGGCTTCTATGAG-3′ (F), 5′-CGCTGCTCTTCTATAGGAACTTG-3′ (R).

### Animal experiments

Animal experiments were conducted with the approval of the Animal Ethics Committee of Renmin Hospital of Wuhan University. Male BALB/c nude mice (4 weeks) from Shulaibao (Biotechnology Co., LTD., Wuhan, China) were raised in a specific pathogen free environment. 1 × 10^7^ cells were suspended in 150μL PBS subcutaneously to establish the xenograft model of mice (6 cells per group). The tumors were resected 6 weeks later to calculate its volume.

In *vivo* metastasis tests, pulmonary metastasis was conducted. The cells were injected into nude mice through the caudal vein. Three weeks later, the mice were sacrificed, the lungs were obtained, and hematoxylin–eosin staining (H&E) was performed to count metastatic nodules.

For activation experiment of SC-79 in *vivo*, 10 days after tumor formation, HCCC9810-AURKB -KD mice were given SC-79 (intraperitoneal injection, 40 mg/kg body weight, once a day, for 10 days), which were set as the experimental group, and the control group was given normal saline. For inhibition experiment of MK-2206 in *vivo*, 10 days after tumor formation, RBE AURKB-OE mice were fed with MK-2206 (oral administration, 240 mg/kg body weight, once every two days, 10 days of administration), which were set as the experimental group, and the control group was fed with normal saline.

### Statistical analysis

All data were indicated as mean ± standard deviation analyzed by GraphPad Prism v8.4.0 and SPSS v25.0 (IBM). All experiments were conducted at least three times. Experimental data were. Statistical differences were determined by the unpaired student t-test or one-way analysis of variance. P < 0.05 was considered statistically significant, and the results were: * P < 0.05, ** P < 0.01.

## Results

### AURKB expression was frequently upregulated in ICC, and AURKB overexpression was associated with poor clinical outcomes

**Fig. 1:**
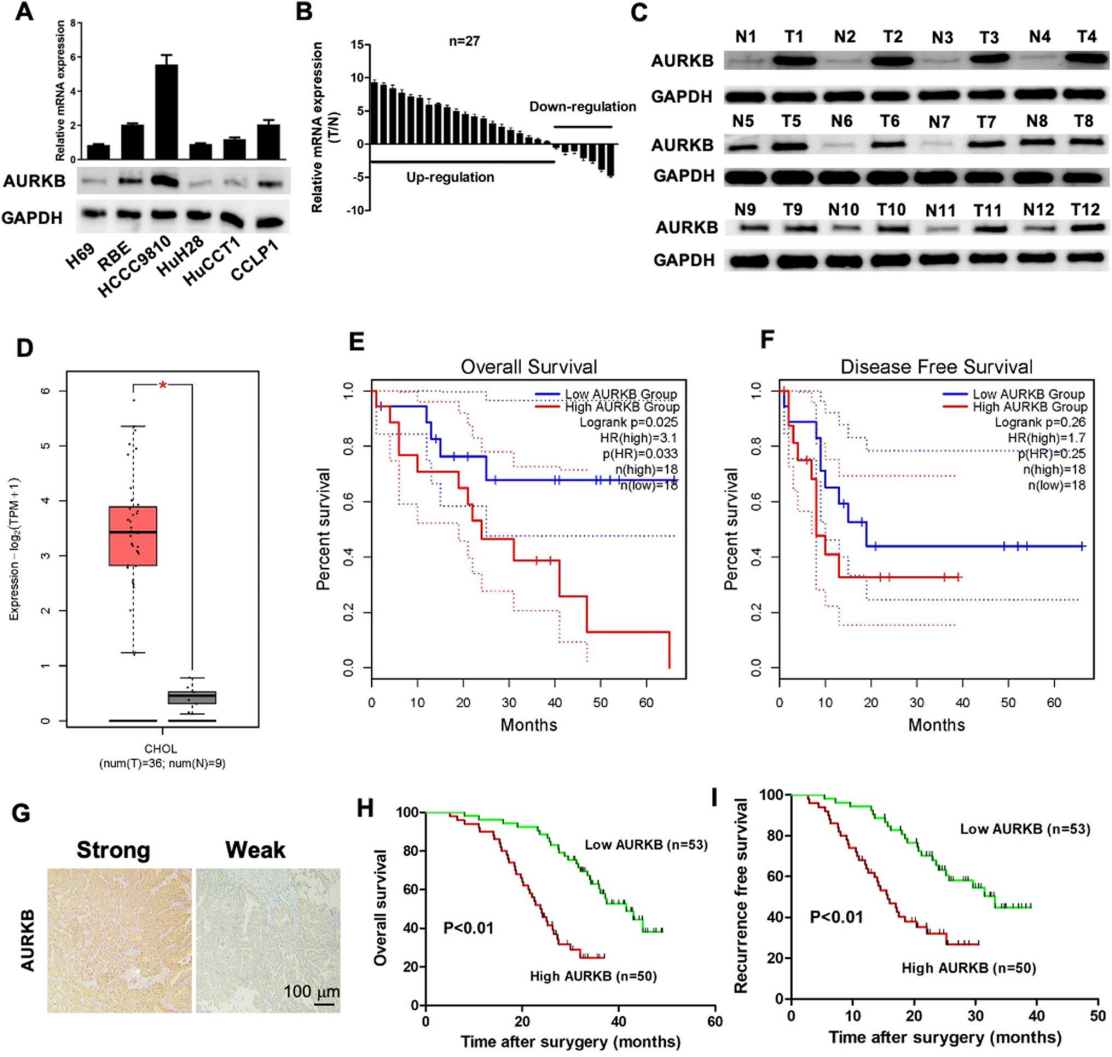
AURKB expression was frequently upregulated in ICC, and AURKB overexpression was associated with poor prognosis. **A** mRNA and protein expression of AURKB in H69 cholangiocytes and ICC cell lines; **B** Comparison of mRNA expression of AURKB in ICC and paracancer tissues; **C** AURKB protein expression in ICC and paracancer; **D** Comparison of mRNA expression in ICC and paracancer tissues in TCGA database; **E** ICC patients AURKB overall survival analysis based on TCGA database; **F** AURKB disease free survival (DFS) analysis of ICC patients with TAGC database; **G** Representative immunohistochemical maps of weak and strong positives in ICC tissue microarrays; **H** OS survival analysis of AURKB in tissue microarrays ICC patients; **I** DFS survival analysis of AURKB in tissue microarrays ICC patients

In the present study, it observed that the protein and mRNA levels of AURKB were high expression in cholangiocarcinoma cells with high metastasis potential (HCCC9810) and low expression in cholangiocarcinoma cells with low metastasis potential (RBE and Huh28) (Fig. 1A, Additional file 1: Fig. S1A). In addition, qRT-PCR results of 27 ICC tissue samples indicated that AURKB level was frequently upregulated in ICC tissues (Fig. 1B). Western blotting also observed similar results, with AURKB protein expression significantly increased in ICC tissues (Fig. 1C, Additional file 1Fig. S1B). GEPIA analysis based on TCGA database showed that AURKB expression was elevated in cholangiocarcinoma (Fig. 1D), and up-regulated AURKB expression was associated with poor overall survival (OS) (Fig. 1E), while disease free survival (DFS) between the AURKB high expression group and the low expression group were not statistically significant (Fig. 1F). Compared with the AURKB intensity in surrounding tissue and control livers as well as ICC tissues with low metastasis potential, AURKB protein expression was increased in ICC tissues with high metastasis potential (Fig. 1G, Additional file 2: Fig. S2). Tissue microarrays survival analysis of 103 ICC patients showed that up-regulated AURKB expression was associated with poor OS and closely correlated with recurrence free survival (RFS) (Fig. 1H, I).

To investigate the relationship between AURKB and clinical characteristics, statistical analysis was performed. It was found that AURKB expression was correlated with serum CA19-9 level (P = 0.006), tumor number (P = 0.008), and lymph node metastasis (P = 0.023) (Table 1). In order to investigate the prognostic significance of AURKB, multivariate Cox regression analysis was performed on recurrence and OS, and it was found that AURKB was the independent prognostic factor for recurrence and OS in ICC patients (Table 2). These results suggest that AURKB expression is frequently upregulated in human ICC and is associated with poor clinical outcomes.

**Table 2:** Multivariate cox proportional regression analysis of prognostic factors in 103 patients

Variables	Recurrence		Overall survival	
	HR (95% CI)	*P*	HR (95% CI)	*P*
CA19-9, ng/ml (> 37versus ≤ 37)	1.18 (0.70–1.98)	0.531	1.16 (0.49–2.74)	0.744
Tumor number (multi versus single)	1.09 (0.47–2.51)	0.838	1.35 (0.83–2.17)	0.225
Lymph node metastasis (yes versus no)	1.68 (0.98–2.91)	0.062	2.048 (0.81–5.1)	0.128
TNM stage (II-III versus I)	1.28 (0.72–2.28)	0.406	1.57 (0.693–3.58)	0.278
AURKB (high versus low)	2.71 (1.28–5.75)	**0.009**	2.58 (1.21–5.49)	**0.013**

CA19-9, carbohydrate antigen 19–9; HR, hazard ratio

### AURKB promoted ICC cell proliferation, cell cycle, migration, invasion and induced EMT in vitro

**Fig. 2:**
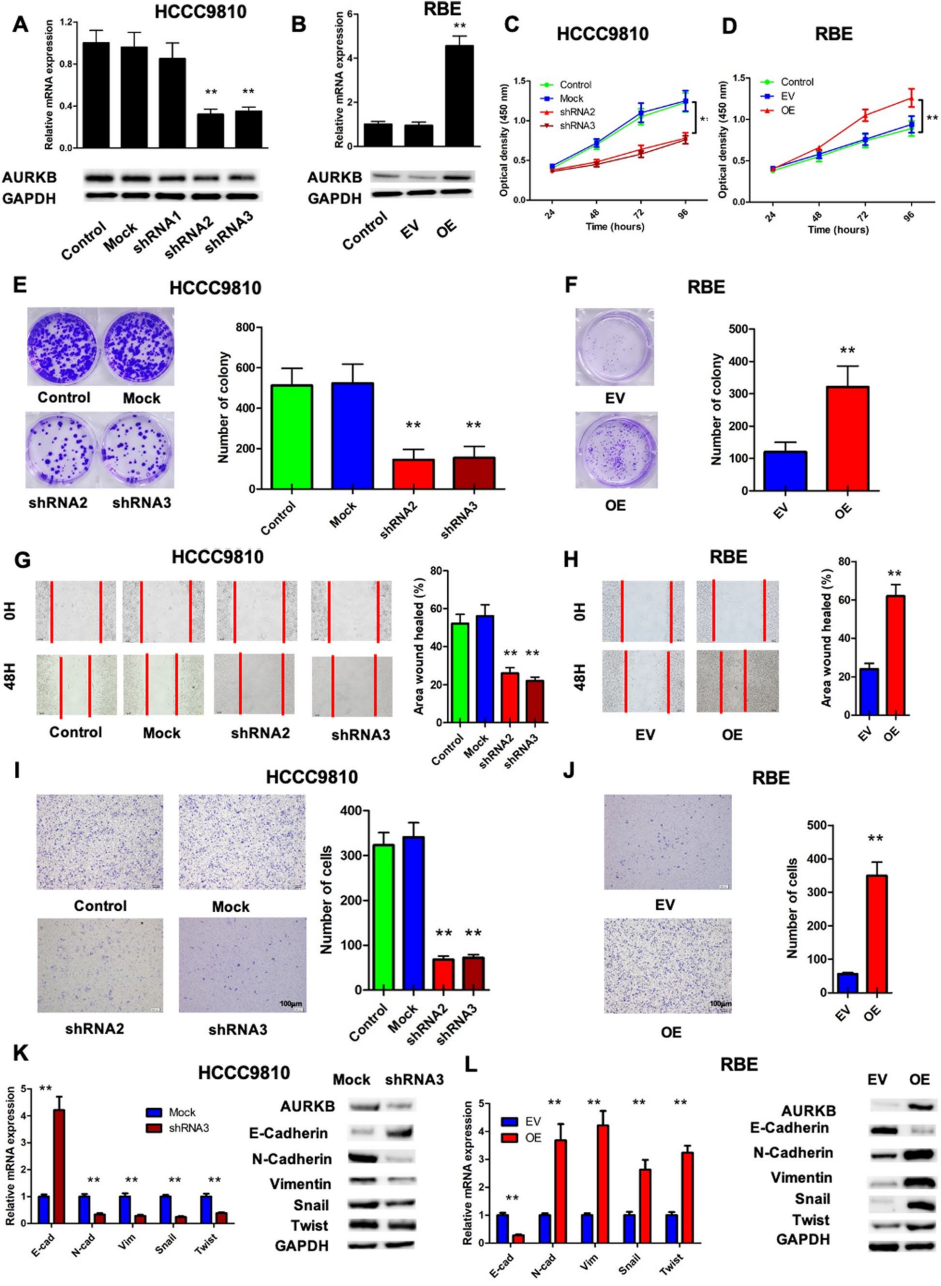
AURKB promoted ICC cell proliferation, migration, invasion and induced EMT in *vitro*. **A** Knockdown efficiency of AURKB gene in HCCC9810 cell line detected by qRT-PCR and western blotting; **B** AURKB overexpression efficiency in RBE cell line detected by qRT-PCR and western blotting; **C** and **D** Cell proliferation after AURKB gene knockdown in HCCC9810 cell line **C** and AURKB gene overexpression in RBE cell line **D**; **E** Colony forming of HCCC9810 cell line after AURKB knockdown; **F** Colony forming of RBE cell line after AURKB overexpression; **G** Wound healing level of HCCC9810 cell line after AURKB knockdown; **H** Wound healing level RBE cell line after AURKB overexpression; **I** Invasion level of HCCC9810 cell line after AURKB knockdown; **J** Invasion level of RBE cell line after AURKB overexpression; **K** Expression of EMT-related marker mRNA and protein in HCCC9810 cell line after AURKB gene knockdown; **L** Expression of EMT-related marker mRNA and protein after AURKB gene overexpression in RBE cell line. * P < 0.05, ** P < 0.01

To explore the function of AURKB in the proliferation and tumorigenicity of ICC cell, the overexpression or knockdown AURKB were conducted with the lentivirus transfection. RBE cells with low AURKB expression were selected for overexpression, and HCCC9810 cells with high AURKB expression were used for knockdown assay. After transfection, shRNA2 and shRNA3 significantly decreased AURKB expression in HCCC9810 cells (Fig. 2A, Additional file 1 Fig. S1C), so shRNA2 and shRNA3 were selected for further experiments. Moreover, AURKB was overexpressed in RBE cells (Fig. 2B, Additional file 1Fig. S1D). Analysis of CCK8 proliferation assay showed that AURKB silencing inhibited HCCC9810 cell proliferation, while AURKB overexpression had the opposite effect and promoted RBE cell proliferation (Fig. 2C, D). Similar to its effect on cell proliferation, in the colony formation experiment, AURKB silencing significantly inhibited the colony number of RBE cells compared to the control group (Fig. 2E), and AURKB overexpression resulted in colony increase in HCCC9810 cells (Fig. 2F). Subsequently, AURKB knockdown inhibited the G1/S and S/G2 transition in HCCC9810 cells, whereas AURKB overexpression enhanced the transition in RBE cells (Additional file 3: Fig. S3A, B). Wound healing assay was performed to investigate the migration ability of RBE and HCCC9810 cells. The results showed that compared with the control group, the wound healing area of HCCC9810 cells was significantly reduced by AURKB knockdown, which inhibited the migration of tumor cells, while the overexpression of AURKB had the opposite effect and promoted the migration of RBE cells (Fig. 2G, H). The invasion assay indicated that the number of invading cells decreased significantly when AURKB was silenced, while the number of invasive cells with AURKB overexpression raised significantly, indicating that AURKB promoted ICC invasion (Fig. 2I, J).

Mesenchymal transformation is invertible dynamic process in which the epithelial cells gradually attain the functional and structural characteristics of the mesenchymal cells. More specifically, EMT is a critical step in the initiation of tumor metastasis events and is achieved for tumor cells to migrate and invade from the primary tumor. To investigate whether AURKB regulates EMT processes, the expression of epithelial and mesenchymal related makers was examined. which declared that epithelial marker E-cadherin was raise and mesenchymal markers N-cadherin and Vimentin were declined in AURKB HCCC9810 cells compared with control cells (Fig. 2K, Additional file 1Fig. S1E). Conversely, AURKB overexpression restrained the expression level of epithelial markers and promoted the expression level of mesenchymal markers in RBE cells (Fig. 2L, Additional file 1Fig. S1F). Thus, these data suggest that AURKB induces EMT processes in ICC.

### AURKB promoted ICC growth and metastasis in vivo

**Fig. 3:**
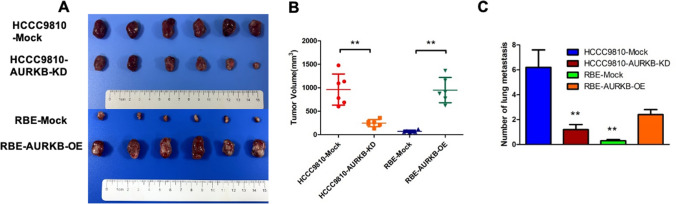
AURKB promoted ICC growth and metastasis in *vivo*. **A** Subcutaneous tumor formation in nude mice after AURKB knockdown and overexpression; **B** Subcutaneous tumor volume in nude mice after AURKB gene knockdown and overexpression; **C** Comparison of lung metastasis in nude mice after AURKB knockdown and overexpression. ** P < 0.01

To investigate the effect of AURKB in tumorigenesis in *vivo*, a mouse subcutaneous xenograft model was established (n = 6/group). The treated cholangiocarcinoma cells and the control cells were injected into different wings of nude mice. After 6 weeks, tumor volume lessened in the HCCC9810 cell group with AURKB knockdown and increased in mice inoculated with AURKB overexpressed RBE cells (Fig. 3A, B). To investigate the effect of AURKB in *vivo* metastasis, a lung metastasis (n = 10/group) model was established. Stable transfected cell lines were had injection into the caudal vein of nude mice and the statement of metastatic nodules in the lungs was surveyed. After 6 weeks, the number and incidence of nodules in HCCC9810 cells with AURKB knockdown significantly decreased compared with the control, while that of nodules in RBE cells with AURKB overexpression significantly increased (Fig. 3C). These data demonstrate that AURKB is involved in ICC cell growth and metastasis in *vitro* and in *vivo*.

### AURKB induced ICC EMT by activating PI3K/AKT pathway in vitro and in vivo

**Fig. 4:**
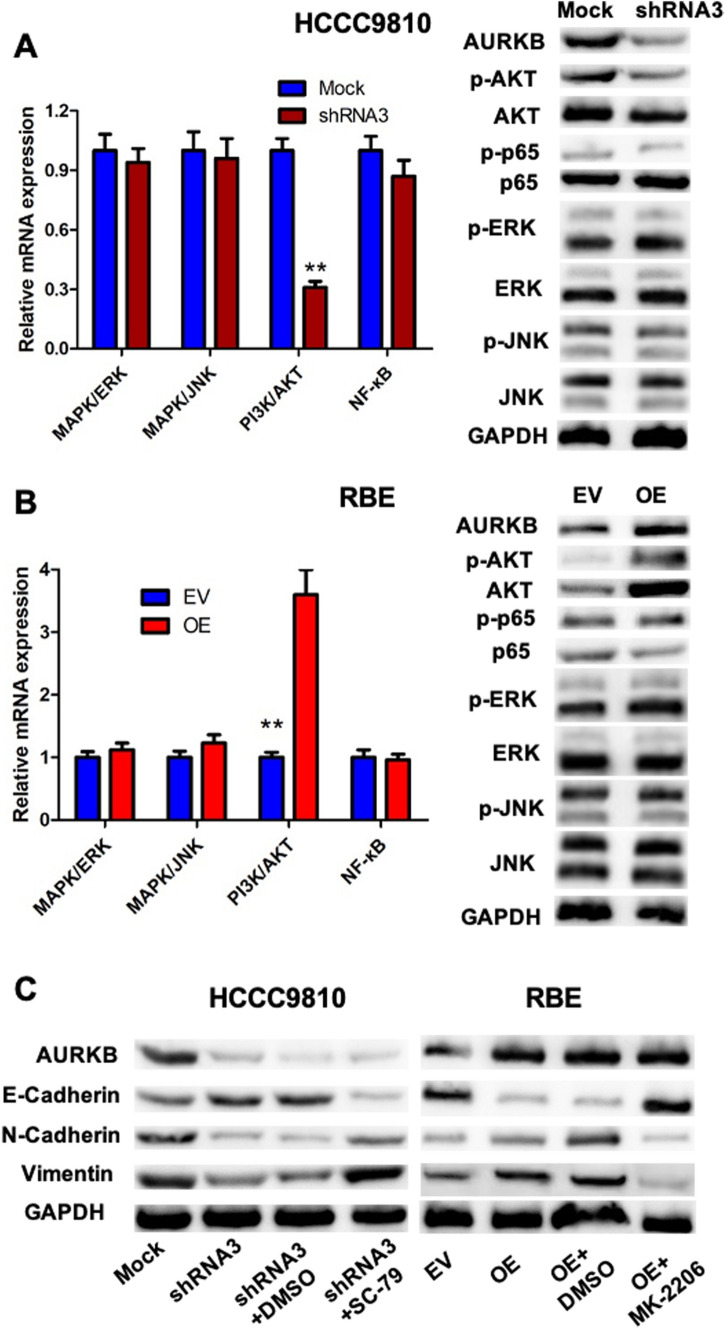
AURKB induced ICC EMT by activating PI3K/AKT signaling pathway. **A** Changes of major EMT-related pathway mRNAs and proteins in HCCC9810 cell line after AURKB knockdown; **B** Changes in mRNAs and proteins of major EMT-related pathways after AURKB overexpression in RBE cell line; **C** Rescue assays of AKT activity after AURKB knockdown and overexpression. ** P < 0.01

Since AURKB induced ICC metastasis, we aimed to ascertain the mechanism of AURKB regulating EMT. Transcriptional program switching in EMT is induced by various signaling pathways, such as NF-κB, PI3K/AKT, JNK, and ERK, which are activated by a variety of dynamic stimuli from the local microenvironment that promote cancer metastasis [23]. To investigate whether AURKB regulates EMT through EMT-related pathways, we examined mRNA and protein changes in major EMT-related pathways. It demonstrated that compared with control cells, p-AKT was down-regulated in AURKB HCCC9810 cells, while p-p65, p-ERK, and p-JNK were not significantly changed (Fig. 4A, Additional file 1Fig. S1G). On the contrary, p-AKT was up-regulated after AURKB overexpression in RBE cells, while p-p65, p-ERK, and p-JNK were not significantly changed as in AURKB knockdown cells (Fig. 4B, Additional file 1Fig. S1H). To further demonstrate that AURKB regulates ICC EMT through activating the PI3K/AKT pathway, HCCC9810 cells were treated with AKT agonist SC-79, the proteins of EMT-related markers were detected by western blotting. Agonist SC-79 reversed upregulation of E-cadherin and downregulation of N-cadherin and Vimentin in AURKB HCCC9810 cells (Fig. 4C, Additional file 1Fig. S1I). Similarity, the AKT inhibitor MK2206 had reversion the E-cadherin downregulation and the upregulation of Vimentin and N-cadherin in RBE cells with AURKB overexpression (Fig. 4C, Additional file 1: Fig. S1J). Moreover, activation experiment of SC-79 in *vivo* revealed that SC-79 promoted the tumor growth and lung metastasis of HCCC9810- AURKB-KD mice, whereas inhibition experiment of MK-2206 in *vivo* showed that MK-2206 inhibited the tumor growth and lung metastasis of RBE AURKB-OE mice (Additional file 4: Fig. S4A–C). The above evidences suggest that AURKB regulates ICC cell EMT by stimulating PI3K/AKT signaling pathway in *vitro* and in *vivo*.

## Discussion

AURKB is a central regulator of chromosome separation and cytokinesis and is abnormally expressed in various cancer cells [8]. Nie et al. [12] reported that AURKB promoted the proliferation of gastric cancer cells in *vitro* and in *vivo*. Yang et al. [24] reported that AURKB accelerates the tumorigenesis and carboplatin resistance by mediating the ERK pathway in neuroblastoma cells. In addition, increased AURKB expression in tumor tissues of patients with breast cancer [25] and metastatic colorectal cancer [26] were also found to be significantly associated with reduced survival. However, the function of AURKB in human ICC remains unclear. In this investigation, the role and mechanism of AURKB in human ICC progression were identified for the first time.

Through the GEPIA database, AURKB was found to be overexpressed in ICC. Furthermore, it was also confirmed that the expression of AURKB was raised in ICC tissues and cell lines. Therefore, we proposed that AURKB has an influence on the progression of ICC. Moreover, GEPIA survival analysis showed that AURKB overexpression was related to the poor prognosis, and this was consistent with previous studies [27]. Tissue chip survival analysis of 103 ICC patients showed that upregulated AURKB expression was associated with poor OS and strongly correlated with poor RFS. We also analyzed the correlation between AURKB expression and clinicopathological parameters in 103 patients and found that AURKB expression was associated with serum CA19-9 level, tumor number, and lymph node metastasis. Moreover, multivariate Cox regression analysis revealed that AURKB was the independent prognostic factor for recurrence and OS in ICC patients. Hence, AURKB was confirmed as a biomarker of novel oncogene and prognostic for ICC.

In order to investigate the pathophysiological and clinical significance of AURKB in ICC and its potential mechanism, the gain- and loss-function experiments were conducted. The results showed that down-regulated inhibition and overexpression of AURKB boosted the proliferation, cell cycle, invasion, migration and metastasis of ICC in *vivo* and in *vitro*. To better explore the latent mechanism of AURKB promoting ICC progression, we conducted a sequence of experiments to testify that AURKB discrepant expression can affect the tumorigenicity and proliferation of ICC cells, as well as influence EMT. EMT has been indicated to significantly facilitate the metastasis of epithelial-derived cancers, including cholangiocarcinoma [28]. We hypothesize that AURKB promotes the metastasis of ICC by modulating EMT. It was demonstrated that AURKB could influence mRNA and protein expression levels of EMT markers in ICC cell lines through in *vitro*. EMT is induced by a variety of signaling pathways, by screening these EMT-related signaling pathways, we found that AURKB overexpression or knockdown significantly affected the protein and mRNA levels of PI3K/AKT signaling. Afterwards, in order to find the effect of AURKB on PI3K/AKT signaling pathway, we performed AKT activity recovery assays after AURKB knockdown and overexpression in *vivo* and in *vitro*, further confirming that AURKB promotes ICC EMT through PI3K/AKT signaling pathway.

In this study, we focused on the activation of EMT by AURKB through the PI3K/AKT axis to promote ICC progression. Nevertheless, the regulation of AURKB expression plays a crucial role. Previous study reported that AURKB overexpression in ICC is associated with p53 [29]. In addition, cyclin dependent kinase 7 (CDK7) regulates AURKB and promotes cell growth, cell cycle, migration, and invasion [30]. TP53 was found to have a high mutation frequency in multiple datasets of ICC using the online database cBioPortal (http://www.cbioportal.org). Interestingly, AURKB had almost no mutation (Additional file 5: Fig. S5). This indicates that AURKB protein expression may not be related to AURKB gene mutation. However, the regulation mode of AURKB expression has not been clarified, and further studies are needed to determine the regulation mode of AURKB expression.

In conclusion, our study suggested that AURKB plays a pivotal role in the progression of ICC via the PI3K/AKT axis in *vivo* and in *vitro*. This study laid a foundation for comprehending the mechanism of ICC and supplied new prognostic biomarkers for ICC.

## Supplementary Information

**Additional file 1: Figure S1.** Quantification of western bolts. **A** Quantification of protein expression of AURKB in H69 cholangiocytes and ICC cell lines; **B** Quantification of AURKB protein expression in ICC and paracancer; **C** Quantification of AURKB protein expression in HCCC9810 cell after AURKB knockdown; **D** Quantification of AURKB protein expression in RBE cell after AURKB overexpression; **E** Quantification of EMT-related marker protein expression in HCCC9810 cell line after AURKB gene knockdown; **F** Quantification of EMT-related marker protein expression after AURKB gene overexpression in RBE cell line. **G** Quantification of major EMT-related pathway proteins expression in HCCC9810 cell line after AURKB knockdown; **H** Quantification of major EMT-related pathways proteins expression after AURKB overexpression in RBE cell line; **I** and **J** Quantification of major EMT-related pathways proteins expression in rescue assays of AKT activity after AURKB knockdown **I** and overexpression **J**. ** P<0.01.

**Additional file 2: Figure S2.** IHC showed AURKB intensity in tissues surrounding ICC patients and in control liver tissues.

**Additional file 3: Figure S3.** AURKB promoted ICC cell cycle. **A** AURKB knockdown inhibited the G1/S and S/G2 transition in HCCC9810 cells; **B** AURKB overexpression enhanced the G1/S and S/G2 transition in RBE cells.

**Additional file 4: Figure S4.** Experiments of AKT agonists and inhibitors in *vivo*. **A** and **B** Activation experiment of SC-79 in *vivo* revealed that SC-79 promoted the tumor growth of HCCC9810-AURKB-KD mice, whereas inhibition experiment of MK-2206 in *vivo* showed that MK-2206 inhibited the tumor growth of RBE AURKB-OE mice; **C** Activation experiment of SC-79 in *vivo* revealed that SC-79 promoted the lung metastasis of HCCC9810-AURKB-KD mice, whereas inhibition experiment of MK-2206 in *vivo* showed that MK-2206 inhibited the lung metastasis of RBE AURKB-OE mice.

**Additional file 5: Figure S5.** The mutation frequencies of TP53 and AURKB were retrieved based on the online database cBioPortal.

## Data Availability

All data included in this study are available upon request by contact with the corresponding author.
