# Supplementary figures and images for: AURKB activates EMT through PI3K/AKT signaling axis to promote ICC progression
Source: Discov Oncol. 2023 Jun 15;14:102. doi: 10.1007/s12672-023-00707-1 (PMC10272048; doi:10.1007/s12672-023-00707-1)

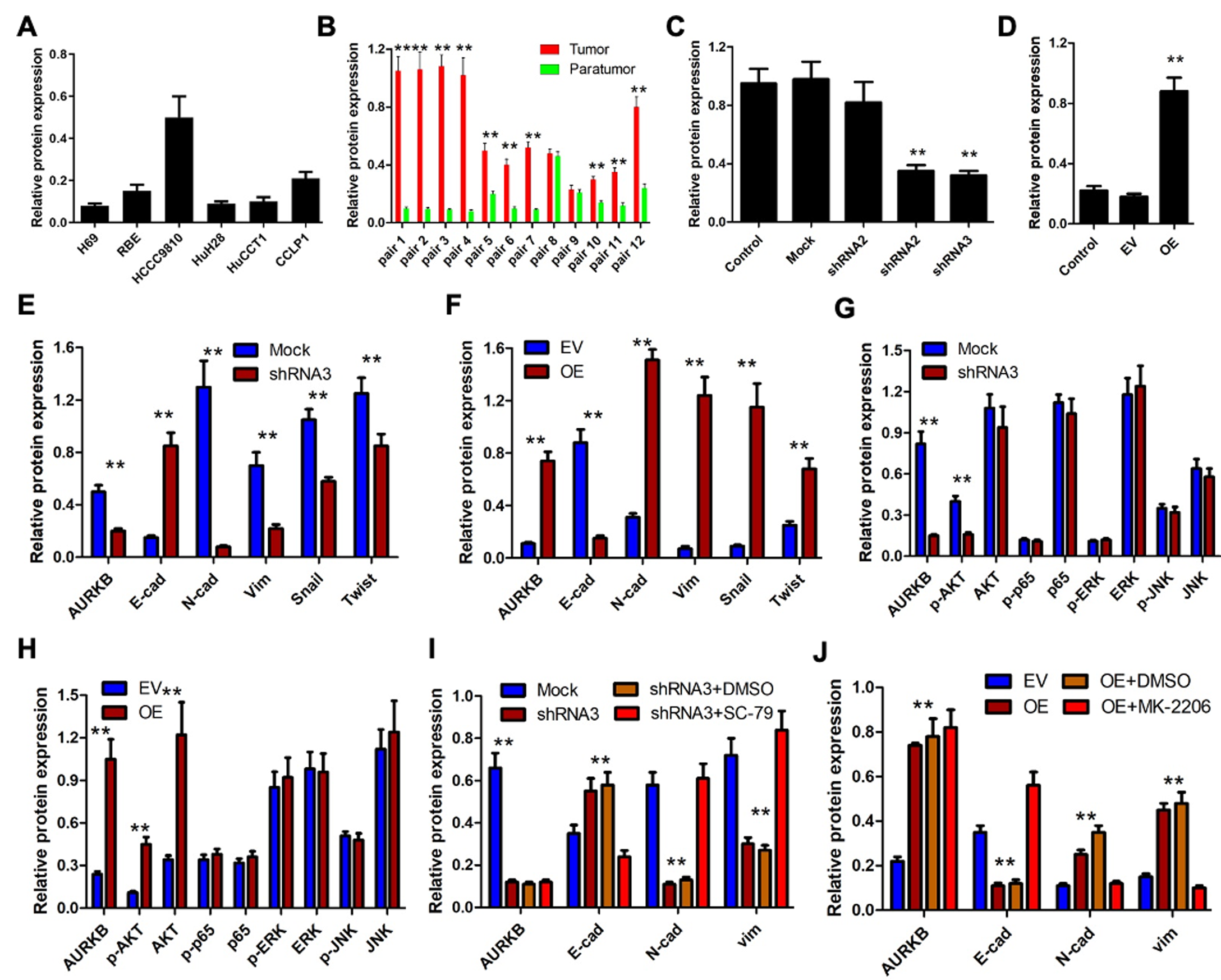

Supplement: Supplementary file 1 — Additional file 1: Figure S1. Quantification of western bolts. A Quantification of protein expression of AURKB in H69 cholangiocytes and ICC cell lines; B Quantification of AURKB protein expression in ICC and paracancer; C Quantification of AURKB protein expression in HCCC9810 cell after AURKB knockdown; D Quantification of AURKB protein expression in RBE cell after AURKB overexpression; E Quantification of EMT-related marker protein expression in HCCC9810 cell line after AURKB gene knockdown; F Quantification of EMT-related marker protein expression after AURKB gene overexpression in RBE cell line. G Quantification of major EMT-related pathway proteins expression in HCCC9810 cell line after AURKB knockdown; H Quantification of major EMT-related pathways proteins expression after AURKB overexpression in RBE cell line; I and J Quantification of major EMT-related pathways proteins expression in rescue assays of AKT activity after AURKB knockdown I and overexpression J. ** P<0.01. [file 12672_2023_707_MOESM1_ESM.tif]

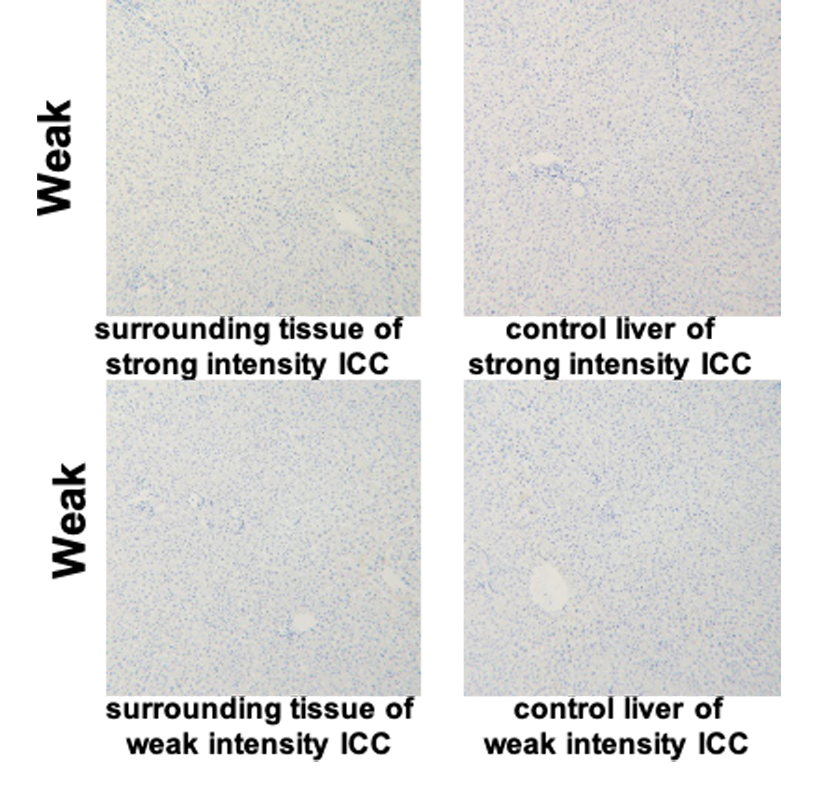

Supplement: Supplementary file 2 — Additional file 2: Figure S2. IHC showed AURKB intensity in tissues surrounding ICC patients and in control liver tissues. [file 12672_2023_707_MOESM2_ESM.tif]

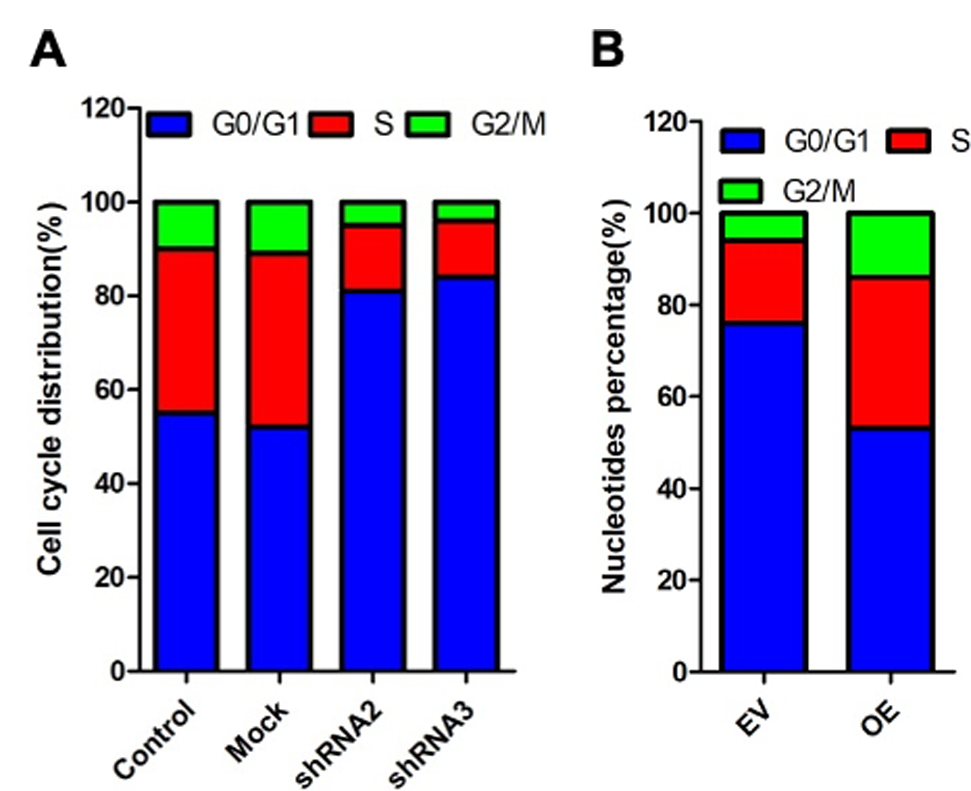

Supplement: Supplementary file 3 — Additional file 3: Figure S3. AURKB promoted ICC cell cycle. A AURKB knockdown inhibited the G1/S and S/G2 transition in HCCC9810 cells; B AURKB overexpression enhanced the G1/S and S/G2 transition in RBE cells. [file 12672_2023_707_MOESM3_ESM.tif]

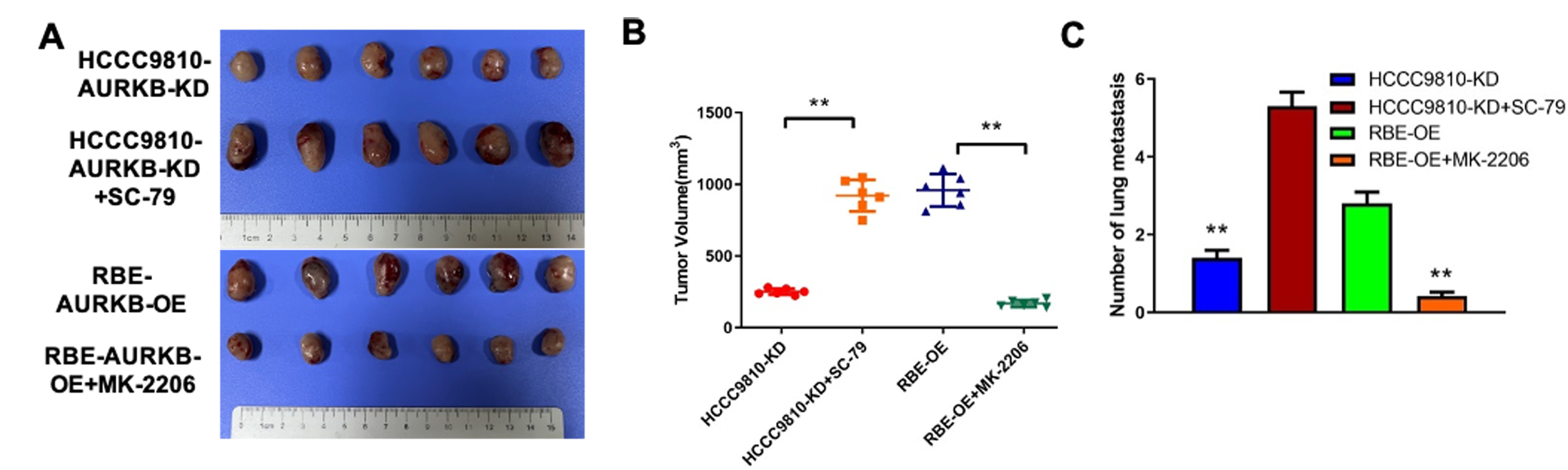

Supplement: Supplementary file 4 — Additional file 4: Figure S4. Experiments of AKT agonists and inhibitors in vivo. A and B Activation experiment of SC-79 in vivo revealed that SC-79 promoted the tumor growth of HCCC9810-AURKB-KD mice, whereas inhibition experiment of MK-2206 in vivo showed that MK-2206 inhibited the tumor growth of RBE AURKB-OE mice; C Activation experiment of SC-79 in vivo revealed that SC-79 promoted the lung metastasis of HCCC9810-AURKB-KD mice, whereas inhibition experiment of MK-2206 in vivo showed that MK-2206 inhibited the lung metastasis of RBE AURKB-OE mice. [file 12672_2023_707_MOESM4_ESM.tif]

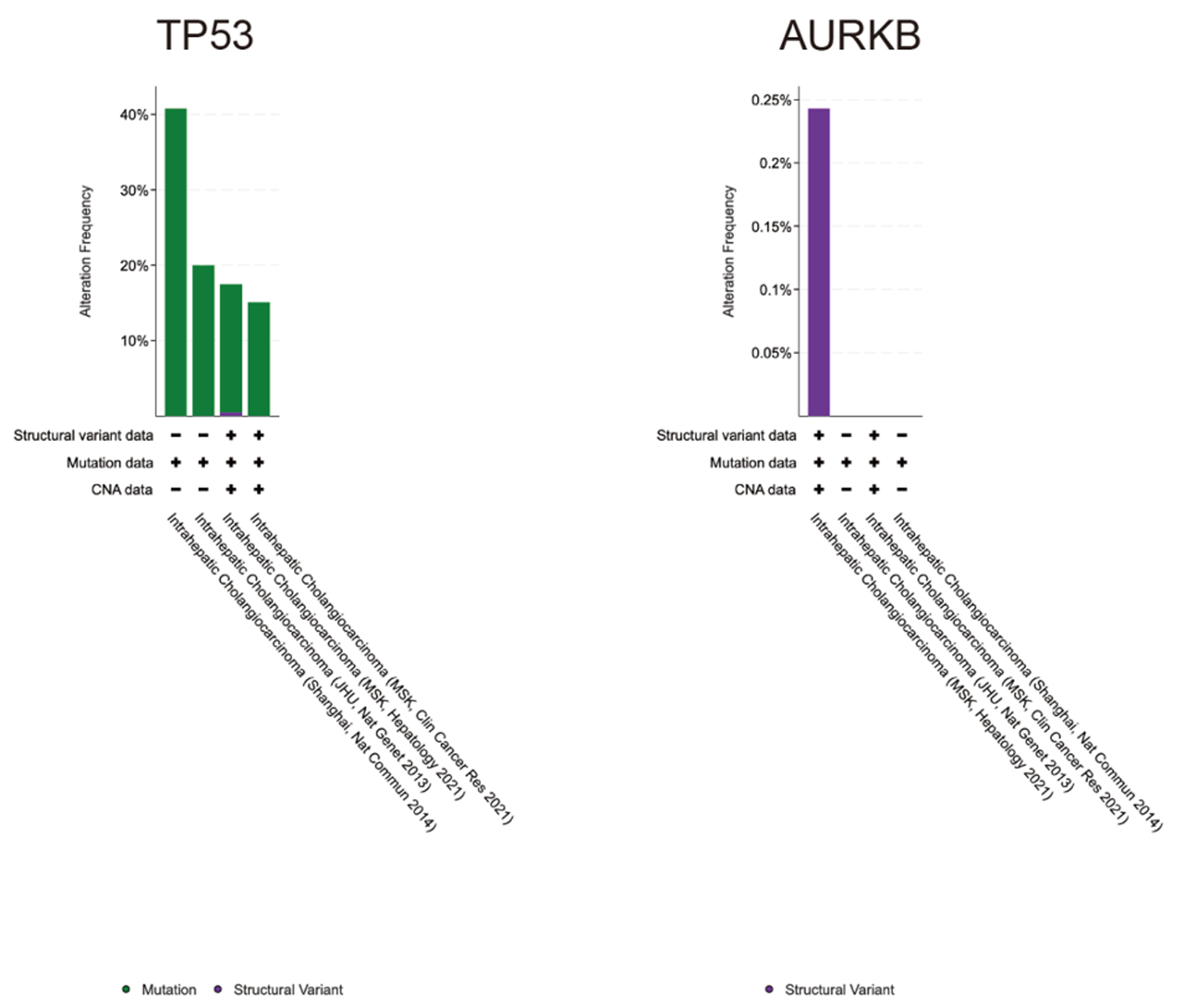

Supplement: Supplementary file 5 — Additional file 5: Figure S5. The mutation frequencies of TP53 and AURKB were retrieved based on the online database cBioPortal. [file 12672_2023_707_MOESM5_ESM.tif]
